# Repositioning of Immunomodulators: A Ray of Hope for Alzheimer’s Disease?

**DOI:** 10.3389/fnins.2020.614643

**Published:** 2020-12-04

**Authors:** Antonio Munafò, Chiara Burgaletto, Giulia Di Benedetto, Marco Di Mauro, Rosaria Di Mauro, Renato Bernardini, Giuseppina Cantarella

**Affiliations:** ^1^Department of Biomedical and Biotechnological Sciences, Section of Pharmacology, University of Catania, Catania, Italy; ^2^Unit of Clinical Toxicology, University Hospital, University of Catania, Catania, Italy

**Keywords:** disease-modifying therapy, clinical trial, drug repurposing, immune response, neuroinflammation

## Abstract

Alzheimer’s disease (AD) is the most common age-related neurodegenerative disorder characterized by cognitive decline and by the presence of amyloid β plaques and neurofibrillary tangles in the brain. Despite recent advances in understanding its pathophysiological mechanisms, to date, there are no disease-modifying therapeutic options, to slow or halt the evolution of neurodegenerative processes in AD. Current pharmacological treatments only transiently mitigate the severity of symptoms, with modest or null overall improvement. Emerging evidence supports the concept that AD is affected by the impaired ability of the immune system to restrain the brain’s pathology. Deep understanding of the relationship between the nervous and the immune system may provide a novel arena to develop effective and safe drugs for AD treatment. Considering the crucial role of inflammatory/immune pathways in AD, here we discuss the current status of the immuno-oncological, immunomodulatory and anti-TNF-α drugs which are being used in preclinical studies or in ongoing clinical trials by means of the drug-repositioning approach.

## Introduction

Alzheimer’s disease (AD) is the most common cause of dementia worldwide, characterized by highest clinical unmet need and huge overall disease burden (Citron, 2010; Lo et al., 2014). AD manifests as a devastating neurodegenerative disorder that, inexorably upset memory, cognitive functions, and the ability to carry out common daily activities (Fischer et al., 2008). The presence in the brain of amyloid beta (Aβ) plaques, composed of Aβ protein and intracellular neurofibrillary tangles, constituted by hyperphosphorylated tau protein, are the two cardinal pathological hallmarks of AD (Querfurth and LaFerla, 2010; Edler et al., 2017; Bulk et al., 2018). Several other hypotheses have been suggested on the pathogenesis of AD, such as neuronal loss, axonal injury, and dysfunction of cholinergic neurotransmission (Hardy and Selkoe, 2002; Haam and Yakel, 2017).

Recent GWAS studies, demonstrating the role of specific genetic variance affecting APP and Aβ processing, showed, at the same time, a tight correlation between immune gene expression and the progression of AD, confirming the crucial role of neuroimmune interactions (Kunkle et al., 2019).

Chronic neuroinflammation is one of the main leitmotiv driving current hypotheses in support of the pathogenesis of AD (Scuderi et al., 2020). Such phenomenon largely derives from aberrant activation of microglia, the brain resident mononuclear phagocytes physiologically involved in central immune surveillance and clearance of pathogens (Burgaletto et al., 2020; Ní Chasaide and Lynch, 2020).

Neuroinflammatory foci in AD localize in close vicinity of Aβ plaques and it is associated with glia activation (Bronzuoli et al., 2016) and the consequent release of inflammatory/immune mediators (Chakraborty et al., 2010), including pro-inflammatory cytokines (Griffin, 2013; Cantarella et al., 2015). In AD, neuroinflammation, instead of being a mere bystander activated by emerging senile plaques and neurofibrillary tangles, substantially contributes to the pathogenesis, synergistically to either Aβ plaques or neurofibrillary tangles (Zhang et al., 2013).

There is also a great deal of evidence suggesting a role of relevance for systemic inflammation in the pathogenesis of AD (Heneka et al., 2015; Ardura-Fabregat et al., 2017; Paouri and Georgopoulos, 2019). Systemic inflammation in AD is associated with an exacerbation of sickness behavior symptoms due to the increased central release of pro-inflammatory cytokines but, importantly, it also acts to accelerate disease progression due to the augmented production of reactive oxygen species and prominent neuronal death (Perry et al., 2010).

In addition to the central nervous system (CNS) resident immune cells, emerging evidence has supported the hypothesis of a relevant role to the peripheral immune system in maintenance of brain homeostasis (Kipnis et al., 2004; Ziv et al., 2006; Filiano et al., 2016) and in disease pathogenesis (Raposo et al., 2014; Baruch et al., 2015; Zenaro et al., 2015; Di Benedetto et al., 2019).

Immune checkpoints are crucial factors in regulating systemic immune homeostasis and tolerance. Selective blockade of some immune checkpoints, such as the Programmed cell death protein-1 (PD-1)/programmed cell death ligand-1 (PD-L1) pathway, enhances anti-tumor immunity by resetting into motion the immune response (Lesokhin et al., 2015). Notwithstanding poor evidence is currently available about the influence of peripheral immune response upon AD brain pathology and related clinical outcomes (Trapnell et al., 2009; Heinz et al., 2010), more recent data suggest that, not only peripheral immunocytes can enter the brain, but also their modulation impacts on its progression (Schwartz and Baruch, 2014; Di Benedetto et al., 2019). Immune modulation in animal models of AD and dementia achieved through treatment with anti-PD-1 or anti–PD-L1 antibodies approaches, resulted in disease modification, manifested by milder pathology paralleled by cognitive improvement (Baruch et al., 2016; Rosenzweig et al., 2019; Castellani and Schwartz, 2020).

Despite the wide number of clinical trials (Cummings et al., 2020), there are currently only four approved pharmacotherapies for AD. Three acetylcholinesterase inhibitors (donepezil, rivastigmine, and galantamine) are recommended as options in the treatment of patients with mild to moderate AD; and memantine, an *N*-methyl-D-aspartate (NMDA) receptor antagonist, is licensed for the management of patients with moderate to severe AD. These agents represent symptomatic treatments, and they do not act as disease modifying drugs, as they only temporarily ameliorate cognitive dysfunction, with relatively modest clinical impact (Cummings et al., 2016).

Drug repositioning represents a valid approach for drug discovery, consisting of finding new indications for currently available drugs used in different clinical settings whose safety and tolerability have already been confirmed (Pillaiyar et al., 2020). For all the above-mentioned reasons, drug repurposing represents a useful tool in searching new treatments for AD (Ihara and Saito, 2020).

Keeping in mind the central role of neuroinflammation, various anti-inflammatory compounds have been re-proposed in AD treatment.

In the wake of several preclinical studies, clinical trials carried out to verify the efficacy of non-steroidal anti-inflammatory drugs (NSAIDs) as curative drugs for AD have failed to show promising results (Ali et al., 2019).

In addition, several drugs indicated for multiple myeloma or leukemia, including daratumumab (anti-CD38 antibody) (Blacher et al., 2015), dasatinib (tyrosine kinase inhibitor) (Zhang et al., 2019), lenalidomide (thalidomide analog; TNF-alpha inhibitor) (He et al., 2013), and sargramostim (granulocyte macrophage colony stimulator) (Kiyota et al., 2018), have been explored for efficacy in AD, based on their immunomodulatory properties assessed in cellular or animal models of AD (Ihara and Saito, 2020).

Considering the key role of the immune/inflammatory response in the development of AD, in the present review we summarize information available concerning the most promising immunomodulatory agents already used in other disease settings for repurposing in AD. In the following sections we will discuss the preclinical evidences underlying possible positioning of each drug in the AD frame. In another section, we will reason about some of the agents which are being studied in ongoing clinical trials.

## Preclinical Evidence

### Immuno-Oncologic Drugs: A Therapeutic Breakthrough in Cancer With a Regard to Neurodegeneration?

Growing body of evidence from multiple studies focus on an inverse epidemiological relationship between AD and cancer. In fact, patients with previous history of cancer have a lower risk of developing AD, otherwise patients suffering from AD show less risk of developing cancer (Rogers et al., 2020).

Research also proved that chemotherapy-treated breast cancer survivors have shown a lower risk of AD compared to healthy control (Monacelli et al., 2017).

**FIGURE 1:**
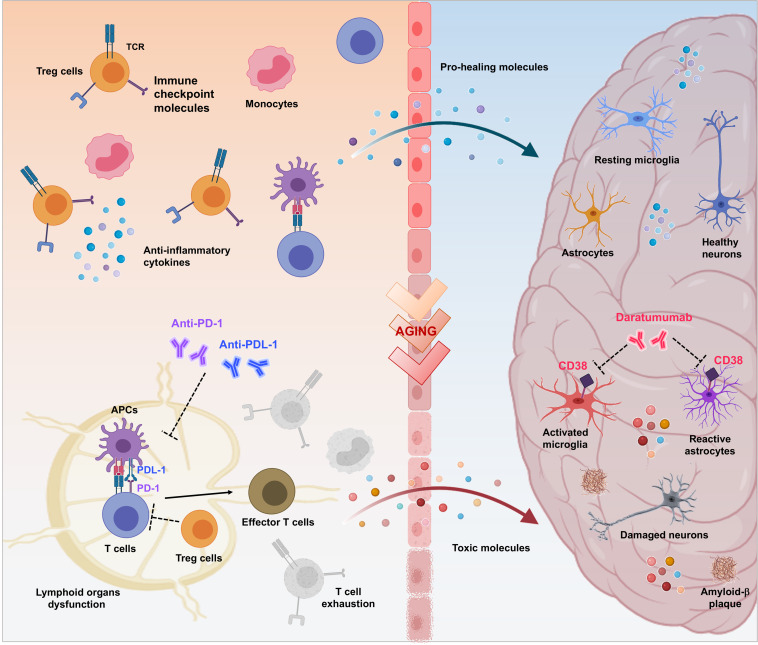
Therapeutic potential of immuno-oncologicals in Alzheimer’s Disease. Immuno-oncological drugs could mark the beginning of a new era for treatment of AD-related neurodegeneration aimed to revitalize the body’s immune-mediated repair mechanisms by addressing multiple pathophysiological factors. As in cancer scenario, controlled trafficking of healing immunocytes to the injured brain could be considered as a mean to overcome immune escape mechanisms and to modify AD progression. Breaking regulatory T cell (Treg)-mediated systemic immune suppression and blocking inhibitory immune checkpoints, such as PD-1/PD-L1 with specific antibodies represents a crucial approach to enhance recruitment of pro-healing immunocytes to the brain parenchyma, culminating in attenuation of the disease-associated pathological features (e.g., dysfunction of lymphoid organs, increase of exhausted T cells, neuroinflammation, production of toxic proteins, neuronal damage and death). Moreover, release of proinflammatory factors that occurs in the brain during aging and AD, results in augmented expression of CD38 in glial cells, amplifying the neurodegenerative cycle. In turn, targeting CD38 with Daratumumab may represent a novel therapeutic approach for modulation of both AD-related neuroinflammation and Aβ production.

Common biological and genetic mechanisms deregulated in opposite directions could explain the phenomenon of mutual protection between AD and cancer (Houck et al., 2018; Okereke and Meadows, 2019). Chronic neuroinflammation related to AD could protects against cancer, otherwise cancer development induces a persistent state of immune tolerance that protects against AD (Rogers et al., 2020). Deep understanding of these mechanisms could represent a trail to follow for designing disease-modifying therapeutic interventions and many anti-cancer agents are the path of repurposing for the treatment of AD. Immunotherapy, helping the immune system to ward off disease, has revolutionized the landscape of cancer treatment and may offer new hope for AD (Figure 1).

#### Immune Checkpoints Inhibitors: PD1 and PD-L1

Programmed cell death protein-1, an inhibitory immune checkpoint receptor (ICR) expressed by immune cells such as T cells, and its broadly expressed ligand PD-L1, have emerged as critical inhibitory signaling pathway that assumes a critical role in maintaining immune homeostasis and self-tolerance preventing autoimmune reaction (Riella et al., 2012; Zhao and Ji, 2019).

In pathological conditions, such as cancer and viral infection, persistent antigen stimulation and inflammation increase the expression level of these ICRs at the T cell surface and its interaction with its ligand on antigen presenting cells (APCs) limit T cell activation, by inducing a hypofunctional state called “exhaustion” (Chamoto et al., 2017; Curdy et al., 2019). This mechanism has emerged as a conceivable therapeutic target for either enhancing or dampening the immune response. Immunosuppressive regulatory T cells (Treg), recruited by abnormal cancer cell chemotactic activity, would have a pivotal role in this process (Baruch et al., 2015).

The development of monoclonal antibodies that target immune checkpoints represents a revolutionary milestone in the field of immuno-oncology (Darvin et al., 2018; Havel et al., 2019) for their ability to modulate the immune response against cancer (Saibil and Ohashi, 2020).

Anti-PD-1/PD-L1 based immunotherapy used as an effective treatment strategy for a wide variety of cancers, including those traditionally considered non-immunogenic (Santarpia et al., 2015), has been recently considered also in animal model of AD as it boosts immune response against the harmful proteins that cause neurodegeneration (Schwartz et al., 2019). Consistently, Cantarella et al. (2003, 2015) have shown that immunoneutralization of Tumor necrosis factor-related apoptosis inducing ligand (TRAIL), a pleiotropic proinflammatory cytokine which also modulates Treg cell functions, results in an improvement of neuroinflammation in a mouse model of AD (Di Benedetto et al., 2019).

Breaking immune tolerance by PD-1 immune checkpoint blockade elicited a stronger interferon (IFN)-γ–dependent systemic immune response, which is followed by the recruitment of monocyte-derived macrophages (MΦ) to the brain, leading to clearance of cerebral Aβ plaques and improved cognition in mice with advanced amyloid pathology (Baruch et al., 2016; Rogers et al., 2020). More recently, it was demonstrated that PD-L1 blockade have efficacy comparable to that of PD-1 blocking in disease modification in AD animal models. In particular, modification of the immunological milieu of the brain mediated by blockade of the PD-1/PD-L1 axis in a mouse model of tau pathology culminates in mitigation of cognitive deficits and cerebral pathology (Rosenzweig et al., 2019).

Consistently with these results, it has been demonstrated that functional PD-1 is expressed in hippocampal neurons and that anti-PD-1 treatment acts also as a neurotherapy potentiating learning and memory by rescue of synaptic transmission and plasticity (Zhao and Ji, 2019).

Conversely, several pharmaceutical companies, that were developing PD-1 antibody inhibitors for other pathologies, pursued this strategy with their own compounds in several Aβ-plaque transgenic models. As expected, PD-1 immunotherapy boosted activation of the peripheral immune system but failed to affect monocyte−derived macrophage infiltration and progression of brain Aβ pathology in three different models of AD (Latta-Mahieu et al., 2018; Obst et al., 2018).

In addition, another research group reports only a modest improvement of locomotor activity without any effect on cognition or tau pathology in a transgenic AD model (Li et al., 2020) by using the same PD-1 checkpoint blockade approach (Baruch et al., 2016; Rosenzweig et al., 2019).

Although the immune checkpoint blockade based-therapy represents a promising therapeutic strategy for AD and age-related dementia, further research is needed before PD-1/PD-L1 based clinical trials are conceived for these disorders.

#### Daratumumab (CD38)

Daratumumab is a first-in-class humanized monoclonal antibody that targets the CD38 epitope approved for multiple myeloma patients who are refractory to conventional therapy (van de Donk, 2018).

Given its role in regulation of neuroinflammatory and brain repair processes, the effect of depletion of CD38, a NAD glycohydrolase expressed by neurons, astrocytes and microglial cells, by daratumumab has been evaluated in the AD context (Guerreiro et al., 2020).

Deletion of CD38 results, in turn, in a significant reduction of Aβ plaque load and soluble Aβ levels and this correlated with improved spatial learning (Blacher et al., 2015).

While direct evidence implicating CD38 in neurodegenerative disorders is still lacking, targeting CD38 may provide a novel therapeutic approach for modulation of both neuroinflammation and Aβ production related to AD.

### Immunomodulating Agents

Immunomodulatory drugs have revolutionized the treatment protocols of various immune-related diseases. These compounds act through modification of the immune response, for example by increasing (immunostimulators) or decreasing (immunosuppressives) the production of serum antibodies (Bascones-Martinez et al., 2014).

**FIGURE 2:**
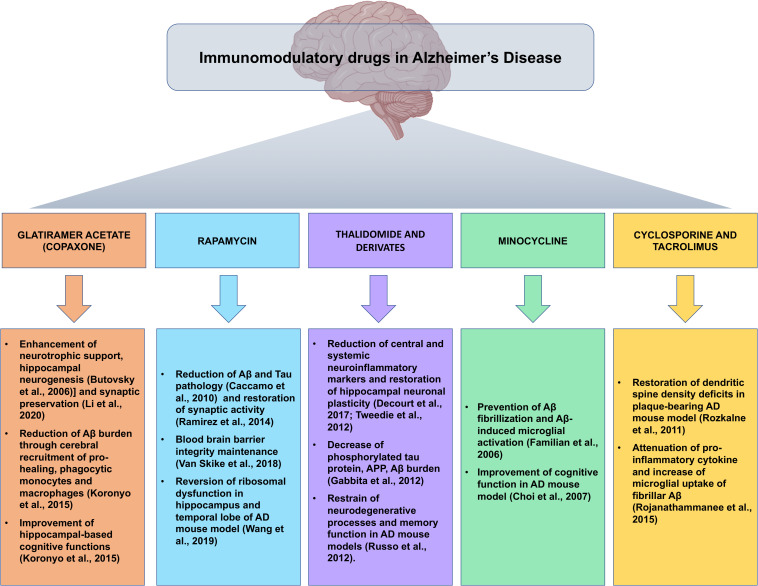
Snapshot of the main preclinical evidences for the implications of immunomodulatory drugs repositioned for Alzheimer’s disease. Summary of the most compelling preclinical evidences of the beneficial effects of immunomodulatory agents in the pathophysiological processes of Alzheimer’s disease, in view of possible future clinical development of immunomodulatory drug-based therapeutic strategies.

Given that the multifactorial pathophysiological mechanism of AD is not restricted to the neuronal compartment, as relevant role has been attributed to the tight interactions of immunological mechanisms within the brain, the repositioning of immunomodulatory drugs could represent an attractive therapeutic strategy in the fight against AD (Figure 2).

#### Glatiramer Acetate (Copaxone)

Glatiramer acetate (GA) (Copaxone) is the first disease-modifying and worldwide-approved drug for the treatment of relapsing-remitting multiple sclerosis (MS), an autoimmune disorder of the CNS (Arnon and Aharoni, 2019). GA is a synthetic analog of myelin basic protein (Scott, 2013), one of the autoantigens implicated in the pathogenesis of MS, which can be used to safely boost T-cell responses without the risk of autoimmune disease, as it weakly cross-reacts with myelin-derived autoantigens (Chen et al., 2015). While the mechanism of action of GA remains a matter of ongoing debate, several evidences suggest that it stimulates Th2 response possibly by suppressing the inflammatory Th1 response (Vieira et al., 2003), increases frequency and function of Treg cells, modulates CD8+ T cells and exerts an immunomodulatory effect on B cells (Lalive et al., 2011).

Immunization of APPSWE/PS1dE9 double-transgenic mice with GA enhances cerebral recruitment of pro-healing, highly phagocytic monocytes (Mo) and MΦ, deeply alleviating cerebral Aβ burden, reducing microgliosis and astrocytosis, finally leading to improved hippocampal-based cognitive functions (Koronyo et al., 2015). Moreover, T cell-based vaccination with GA in the same animal model of AD leads to enhanced neurotrophic support and hippocampal neurogenesis (Butovsky et al., 2006).

Moreover, nasal vaccination with a proteosome-based adjuvant plus GA leads to activation of pro-healing microglia, strongly correlated with a decrease in Aβ fibrils in APP-Tg mice (Frenkel et al., 2005).

Recently, it has been demonstrated both in *in vivo* and *in vitro* studies that GA-stimulated MΦ protect neurons from Aβ-mediated synaptotoxicity through enhanced ability to eliminate Aβ42 oligomers and induce synaptic preservation (Li et al., 2020). Moreover, GA immunomodulation enhanced cerebral recruitment of Mo-derived MΦ and reversed loss of cortical and hippocampal excitatory synapses in mouse models of AD.

Consistently, it has been demonstrated that GA immunization significantly increases expression of hippocampal early growth response protein 1 (Egr1), protein required for synaptic plasticity and memory formation, which was negatively correlated with hippocampal Aβ plaque burden (Bakalash et al., 2011).

GA-based vaccination could provide a new avenue for immune therapy that might prove efficacious in the treatment of AD.

#### Rapamycin

Rapamycin is a macrolide antibiotic and inhibitor of the mechanistic target of rapamycin (mTOR) that exhibits potent anti-cancer and immunosuppressive activity (Law, 2005) originally used to prevent organ transplant rejection (Richardson et al., 2015).

Currently, this drug represents the most effective pharmacological approach for directly targeting hallmarks of the aging process in order to increase lifespan in several animal models.

In addition to its efficacy at mitigating physiological aging, this drug has been shown to have a beneficial effect in models of neurodegeneration and aging, including mouse models of AD (Kaeberlein and Galvan, 2019).

The positive outcomes of rapamycin treatment probably stand up on its ability to rescue molecular pathways associated with aberrant mTOR phosphorylation, responsible to speed up the age-related neurodegenerative process and increase the risk of developing AD (Caccamo et al., 2010).

In fact, it has been demonstrated that restoring mTOR signaling with rapamycin ameliorates Aβ and tau pathology in several mouse models, preserves blood brain barrier (BBB) integrity, restores cerebral blood flow and brain vascular density and rescues cognitive deficits(Lin et al., 2013; Van Skike et al., 2018; Gureev et al., 2020).

Rapamycin is also able to regulate cholesterol biosynthesis, essential for synaptic formation and to reverse ribosomal dysfunction in hippocampus and temporal lobe of APP/PS1 mouse (Wang et al., 2019). In addition, rapamycin protects hippocampal neurons from synaptotoxicity induced by Aβ oligomers by increasing presynaptic activity (Ramírez et al., 2014).

Similarly, chronic treatment with the rapamycin derivative temsirolimus, a recently developed compound used for renal cell carcinoma treatment, promotes autophagic Aβ clearance, reduces neurofibrillary tangle density and attenuates apoptosis in hippocampus, leading to a substantial improvement in spatial learning and memory abilities (Jiang et al., 2014).

Conversely, one study revealed that rapamycin can only prevent, but not rescue, the accumulation of amyloid plaques and tangles, as well as cognitive deficits (Majumder et al., 2011).

Current preclinical data reveals that rapamycin may be valuable for preventing the onset or early AD neuropathology, and, however, cannot represents a treatment option in people with overt clinical signs of dementia. Altogether, it is plausible to propose rapamycin as an agent that, if used in the prodromic stages of AD, would probably demonstrate effectiveness in delaying progression of dementia.

#### Thalidomide and Its Derivatives

Thalidomide and its derivatives, referred to as immunomodulatory imide drugs (IMiDs), are a class of drugs that target the 3′-untranslated region (3′-UTR) of Tumor necrosis factor alpha (TNF-α) mRNA, inhibiting TNF-α cytokine production. Preclinical studies on currently marketed IMiDs, indicate improved BBB permeability and bioavailability when compared to similar anti-inflammatory agents, supporting the concept of their development as drugs for neurological disorders (Jung et al., 2019).

Thalidomide is a potent immunomodulator and a TNF-α inhibitor, originally used for treatment of multiple myeloma and erythema nodosum leprosum (Jung et al., 2019) and evaluated for repurposing across numerous neurological disorders due to its multipotent pleiotropic characteristics.

Chronic thalidomide administration significantly blunts both astrocytes and microglia activation, and Aβ generation in brains of APP23 transgenic mice through inhibition of beta-secretase (BACE1) (He et al., 2013; Decourt et al., 2017).

Moreover, 3,6′-dithiothalidomide (3,6′-DT) effectively lowers TNF-α, nitrite and secreted amyloid precursor protein (sAPP) levels *in vitro* in LPS-activated macrophage-like cells, while it significantly reduces central and systemic TNF-α production, neuroinflammatory markers and restores hippocampal neuronal plasticity in LPS-challenged rats (Tweedie et al., 2012). Chronic 3,6′-DT administration reduces multiple hallmark features of AD, including glia activation, phosphorylated tau protein, APP, Aβ peptide and Aβ-plaque number along cognitive dysfunction in 3×Tg-AD mice, and leads to synaptic preservation (Gabbita et al., 2012; Tweedie et al., 2012). As a matter of fact, 3,6’-DT ameliorates Aβ-induced neuroinflammation and microglial activation, preventing neurodegeneration and improving memory in AD mouse model of stereotaxic intracerebroventricular Aβ1-42 (Russo et al., 2012).

Recently, it has been demonstrated that also Pomalidomide (Pom), an immunomodulatory amino-thalidomide analog, and Pom analog 3,6′-dithioPom (DP), significantly mitigate Traumatic brain injury (TBI)-induced cell death, neurodegeneration, astrogliosis, microglial activation, neuroinflammation and behavioral impairments in TBI which represents a process tightly associated with the later development of dementia (Lin et al., 2020).

Taken together, these preclinical studies using IMiDs have shown promising profiles, indicating a potential for the promotion of this therapeutic class from the bench to clinical trials and eventually, to the bedside of AD patients.

#### Minocycline

Minocycline is a member of tetracycline family antibiotic with anti-inflammatory and immunomodulatory properties, largely used in the treatment of acne vulgaris and various sexually transmitted diseases (Garrido-Mesa et al., 2013). Based upon its ability to cross the BBB and to inhibit microglial cells, minocycline has been regarded as a repurposing candidate for evaluation in AD (Shamim and Laskowski, 2017).

Minocycline prevents Aβ fibrillization and Aβ-induced microglial activation *in vitro* (Familian et al., 2006) leading to attenuation of inflammatory response and microgliosis, as well as to a significant improvement of cognitive deficit (Fan et al., 2007). Similar beneficial effects on cognitive functions were obtained in a Aβ1-42-infused rat model and Tg2576 mice treated intraperitoneally with minocycline (Choi et al., 2007).

Moreover, it has been reported that minocycline has different effects on Aβ plaque deposition depending upon the age of administration, due to its action on microglial function (Fu et al., 2019). In addition, minocycline is able to significantly restrain the early, pre-plaque neuroinflammatory response, and also to reduce APP expression; moreover, it inhibits BACE1 activity in McGill-Thy1-APP mice (Ferretti et al., 2012).

Minocycline is able to reduce microglia reactivity in the dentate gyrus, as well as inducible nitric oxide synthase protein levels and reactivity of Aβ plaque-associated CD11b+ microglia in the hippocampus of APP/PS1 mice (Biscaro et al., 2012).

#### Cyclosporine and Tacrolimus

Inhibitors of calcineurin such as Cyclosporine and Tacrolimus, are immunosuppressive agents used for the prophylaxis of post-transplant organ rejection and to treat autoimmune diseases (Khanna, 2000).

In the AD scenario, these agents downregulate the expression of APP mRNA and protein in primary cultures of neonatal rat astrocytes (Lee et al., 1999).

Furthermore, short-term treatment with tacrolimus ameliorates dendritic spine density deficits in plaque-bearing AD model mice (Rozkalne et al., 2011).

More recently, it has been demonstrated that Tacrolimus significantly attenuated both Aβ- and LPS-stimulated secretion of pro-inflammatory cytokines and increased microglial uptake of fibrillar Aβ *in vitro*, while it led to decreased spleen cytokine levels, microgliosis and Aβ plaque burden in APP/PS1 mice (Rojanathammanee et al., 2015). Cyclosporine and Tacrolimus treatment significantly attenuates Streptozocin-induced biochemical and histopathological alterations and age-related memory deficits. This evidence demonstrates the potential of these agents in cognitive dysfunctions, probably related to their anti-amyloid, anti-oxidative and anti-inflammatory properties (Kumar and Singh, 2017).

### TNF-α Blocking Agents

TNF-α is a potent proinflammatory cytokine that plays a central role in setting into motion and sustaining the inflammatory response.

TNF-α signaling exerts both homeostatic and pathophysiological roles in the CNS.

In the healthy CNS, TNF-α has regulatory functions on synaptic plasticity, control of microglial activation and astrocyte-induced synaptic strengthening, and regulation of glutamatergic transmission (Belarbi et al., 2012; Olmos and Lladó, 2014).

In pathological conditions, microglia release large amounts of TNF-α that represents a critical mediator of neuronal dysfunction and cognitive impairment consequent to chronic neuroinflammation (Olmos and Lladó, 2014).

TNF-α contributes to disease onset and progression in transgenic mouse models of AD (Chang et al., 2017).

Clinical involvement of TNF-α in AD has been evidenced by the observation of elevated TNF-α levels in the plasma and in the cerebrospinal fluid (CSF) of AD patients and by the co-localization of TNF-α with Aβ plaques in the brain, both correlated with disease severity (Steeland et al., 2018).

Several TNF-α–specific monoclonal antibodies (e.g., infliximab, adalimumab) and recombinant fusion proteins (etanercept), often developed for peripheral inflammatory conditions including Crohn’s disease and rheumatoid arthritis, have been tested on AD rodent models using both central and peripheral routes of administration (Chang et al., 2017; Decourt et al., 2017). Treatment with TNF-blocking agents in patients with rheumatic disorders is associated with lower risk for AD development (Zhou et al., 2020).

Nevertheless, limited BBB penetration of these agents is the main drawback for their development (Chang et al., 2017). Thus, peripheral targeting of TNF-α activity and reengineering of the TNF-α inhibitors able to cross BBB represent two methods to reasonably overcome such limitations (Yiannopoulou and Papageorgiou, 2020).

**FIGURE 3:**
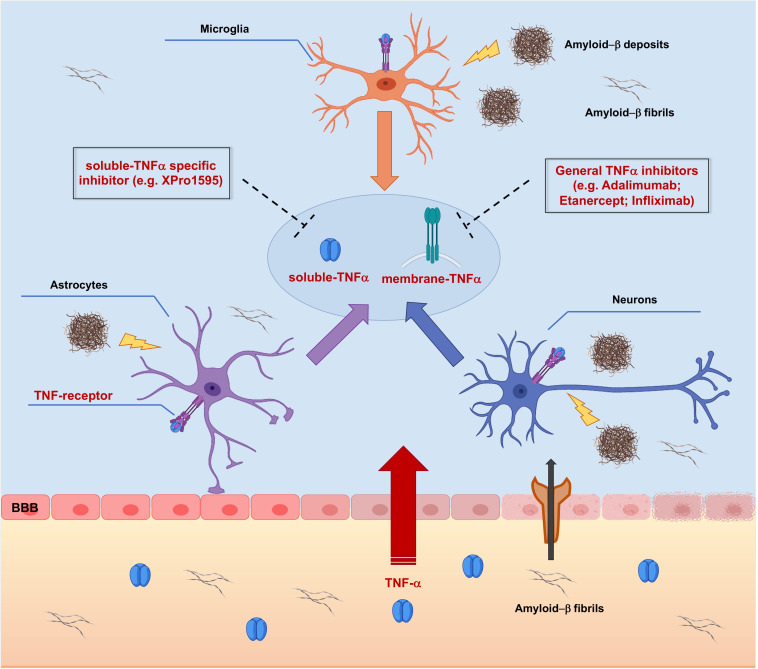
Targeting TNF-α signaling with TNF-α inhibitors in Alzheimer’s Disease pathology. Peripheral Tumor Necrosis Factor-α (TNF-α) enters the brain through the Blood-Brain Barrier (BBB) via transcytosis, upsetting its structural integrity and the permeability. TNF-α affects brain resident cells by binding Tumor Necrosis Factor Receptors (TNFRs), thus triggering activation of different intracellular cascades which redundantly lead to increased release of TNF-α. In addition, TNF-α leads to increased peripheral amyloid-β (Aβ) influx and generation of amyloid deposits into the brain parenchyma, which supplement the local amyloid burden, causing further production of TNF-α. Targeting membrane bound and/or soluble TNFα with small molecule inhibitors could represent a potential effective therapeutic approach acting at earliest steps of the AD-related neuroinflammatory vicious cycle, resulting in neuroprotection.

However, targeting TNF-α synthesis with inhibitors (Figure 3) has also been proposed to have a great potential for the long-term prevention and treatment of AD (Belarbi et al., 2012).

#### Etanercept

Etanercept, a bio-engineered, anti-rheumatoid, anti-TNF-α fusion protein that binds both soluble and membrane-bound forms of TNF-α, has been re-evaluated also as a treatment to hold off central and peripheral immune/inflammatory response in AD (Butchart et al., 2015).

Peripheral administration of etanercept counteracts Aβ-induced memory impairment and attenuates hippocampal levels of TNF in a non-transgenic mouse model of amyloid induced cognitive deficits (Detrait et al., 2014; Chang et al., 2017).

Recent evidence reports that etanercept leads to a decrease of plaques burden and neurofibrillary tangles and improves cognitive outcomes in streptozocin-treated rats, widely used to mimic an AD-like condition in animal models (Kübra Elçioğlu et al., 2015).

Although there are many evidences for the beneficial effect of etanercept, it still remains to be explored whether this drug is able to alter AD-like neuropathology in AD chronic models.

#### Infliximab

Infliximab is a monoclonal antibody against TNF-α approved for treatment of rheumatoid arthritis, Crohn’s disease and other immune-mediated inflammatory disorders (Melsheimer et al., 2019).

Intracerebroventricular injection of Infliximab, beside reducing levels of TNF-α, induced rapid and transient decline in Aβ loads and tau phosphorylation in the APP/PS1 double transgenic mice (Shi et al., 2011).

Recently, it has been also demonstrated that infliximab dramatically improves visual recognition memory impaired by Aβ oligomers and reverses the noxious effect of Aβ on muscarinic acetylcholine receptor–dependent long-term depression of synaptic transmission in Tg2576 mice (Kim et al., 2016; Chang et al., 2017). Kübra Elçioğlu et al. (2015) reported also that infliximab, as demonstrated for etanercept, led to a significant improvement of cognitive functions in rat models of dementia.

Peripheral inhibition of TNF-α with infliximab in the context of arthritis, modulates the amyloid pathology by regulating blood-derived and local brain inflammatory cell populations involved in β-amyloid clearance in the brain of double-transgenic 5XFAD/Tg197 AD/TNF mice that develop amyloid deposits and inflammatory arthritis induced by human TNF-α expression (Paouri et al., 2017).

Despite these favorable evidences, other preclinical studies in mouse AD models with TNF-α inhibitors failed to reproduce such beneficial effects (Giuliani et al., 2009; Chang et al., 2017).

#### Adalimumab

Adalimumab, another anti-TNF-α monoclonal antibody, binds directly to TNF-α or to soluble and membrane-TNF-α receptors blocking ligand-receptor interactions (Scheinfeld, 2005). Adalimumab has demonstrated efficacy and tolerability in patients with a wide range of inflammatory conditions (Lapadula et al., 2014).

Adalimumab treatment leads to significantly attenuated neuronal damage and neuroinflammation, decreased beta secretase-1 protein expression and Aβ1-40 plaques, and to improvement of cognitive functions in Aβ1-40-injected mice (Park et al., 2019; Anwar and Rivest, 2020), supplying a rationale for a hypothesis of clinically meaningful outcomes in patients with AD.

#### XPro1595

XPro1595 is a second-generation TNF-α inhibitor, which, unlike etanercept and other non-selective TNF-α inhibitors, solely targets the soluble form of TNF-α, preserving the neuroprotective transmembrane TNF-α signaling pathways (Steed et al., 2003).

Preclinical XPro1595 evaluation has been reported in three different mouse models of AD. Peripheral administration of the soluble TNF-α inhibitor XPro1595 is able to reduce brain amyloid deposition, age-dependent increase in activated immune cells and to improve synaptic function (Cavanagh et al., 2016; MacPherson et al., 2017). Local administration of XPro1595 leads to reduced pre-plaque Aβ pathology in 3×TgAD mice (McAlpine et al., 2009), and, consistently, it reduces microglia activation and improves synaptic and cognitive functions in aging rats (Sama et al., 2012).

## Clinical Evidences

Based on preclinical data, in recent years, numerous clinical trials have been conducted aimed to deepen the therapeutic potential of the above-mentioned drug classes for AD. However, most have failed to demonstrate promising results, probably because of the still incomplete understanding of the role of neuroinflammation in the development of AD combined to the lack of apposite diagnostic tools to determine stages of the disease (Cummings et al., 2020).

In this section, we report ongoing clinical trials that employ the drug-repositioning method for drug discovery of AD (Table 1).

**TABLE 1:** Ongoing clinical trials that use the drug-repositioning method for drug discovery of AD.

**Drug**	**Phase**	**Duration**	**Number of patients**	**Official Title**	**Primary Outcome Measures**	**ClinicalTrials.gov Identifier**
Daratumumab	Phase 2	24 weeks	15	*An Open-Label, Pilot Study of Daratumumab SC in Patients With Mild to Moderate Alzheimer’s Disease* (DARZAD)	ADAS-cog/11 [Time Frame: 25 weeks] Responder rate defined as improvement of ≥4 points on standard 11-item.	NCT04070378
Rapamycin	Phase 1	8 weeks	10	*Cognition, Age, and Rapamycin Effectiveness Downregulation of the m-Tor Pathway* (*CARPE DIEM*)	Blood brain barrier penetration of RAPA [Time Frame: Change from Baseline to 8 weeks]	NCT04200911
Lenalidomide	Phase 2	18 month	30	MCLENA-1: A *Phase II Clinical Trial for the Assessment of Safety, Tolerability, and Efficacy of Lenalidomide in Patients With Mild Cognitive Impairment Due to Alzheimer’s Disease*	Change in cognition as assessed by the Alzheimer’s Disease Assessment Scale-Cognitive Subscale (ADAS-Cog) total score [Time Frame: 18 months]	NCT04032626
Tacrolimus	Phase 2	12 weeks	12	*A Pilot Open Labeled Study of Tacrolimus to Assess its Effects on Bio-markers of Mild Cognitive Impairment and Alzheimer’s Disease*	CSF biomarkers of target engagement, AD pathology, and neurodegeneration [Time Frame: Baseline and 12 weeks]	NCT04263519
XPro1595	Phase 1	12 weeks	18	*Phase 1b Open-Label, Dose-Identification Study of XPro1595 in Patients With Mild to Moderate Alzheimer’s Disease With Elevated High Sensitivity C-reactive Protein in Blood*	The number and percentage of patients with a treatment-emergent adverse event throughout 12 weeks of treatment with XPro1595	NCT03943264

### Daratumumab (NCT04070378)

Currently Daratumumab is the only monoclonal antibody in study for drug repositioning in AD. The rationale behind the use of this drug lies in its immunomodulatory action against CD38+ cells.

As mentioned above, CD38 is a multifunctional protein with both a receptor and an enzyme-mediated function involved in several important reactions for the physiological neuronal development (Guerreiro et al., 2020).

CD38 expression increases during neuroinflammation and neurodegeneration, suggesting its potential modulating role in brain cells regulation. Experiments on CD38 knockout mice (Roboon et al., 2019), demonstrated a decreased release of pro-inflammatory cytokines and chemokines (Kou et al., 2009), while its overexpression was found after treatment with drugs-induced neuroinflammation (Guerreiro et al., 2020).

Interestingly, CD38 expression on CD8+ T-cells is significantly increased in AD patients as compared with age-matched controls (Gate et al., 2020) and these activated T-cells are able to infiltrate into the CNS exerting toxic effects.

The objective of the clinical trial has been to explore whether treatment with Daratumumab, an agent able to cross the BBB, may have a clinically meaningful effect on patients with mild to moderate AD.

The study includes patients with diagnosis of AD, without a clinical history of other neurological or psychiatric disorders, according to NIA-AA criteria, a MMSE score between 15 and 26 and positive instrumental with MRI and amyloid PET scan, on a stable dose of cholinesterase inhibitor for at least 12 weeks. Patients in treatment with anti-Aβ or anti-tau protein, vaccine with live/live-attenuated bacterial or virus in the latest 3 months, immunosuppressant and corticosteroids in the latest 2 months, anticoagulant, estrogens have been excluded, as well as patients with HCV, HBV, HIV infections or malignancy in the previous 2 years. The primary endpoint is an improvement of at least 4 points at ADAS-cog/11 after 24 weeks of treatment. Secondary endpoints include unchanging or improvement at ADAS-cog/12, MMSE, CDR-SB, ADCOMS after 24 weeks of treatment from baseline. Adverse and serious adverse effects will be assessed after 35 weeks from initial treatment. Study is estimated to be completed within the end of 2021.

### Rapamycin (NCT04200911)

In light of preclinical evidence, rapamycin is an effective inhibitor of AD-related neurodegeneration (Cai et al., 2012). The most important suggested mechanisms include enhancement of autophagy and the consequent increase of the clearance of Aβ aggregates (Santos et al., 2011), and attenuation of tau hyperphosphorylation (Liu et al., 2013).

The combination of these elements supported the running of the study Cognition, Age, and RaPamycin Effectiveness–DownregulatIon of the mTOR-pathway (*CARPE DIEM*), an early phase 1 clinical trial, involving 10 patients, in a single group, finalized to evaluate the effect of oral Rapamycin in older adults with AD and mild cognitive impairment (MCI). It represents an open-label pilot study that, once established the feasibility and safety of the treatment, should constitute an initial proof-of-concept for a larger Phase 2 clinical trial.

In this study, Sirolimus 1 mg has been administered *per os* once a day for 8 weeks. The primary endpoint measures the penetration of Rapamycin across BBB, by means of lumbar puncture at baseline and after the final dose, while the secondary endpoints include changes in AD progression through evaluation of AD biomarkers, as well as cognitive and physical tests.

The 10 patients recruited, between 55 and 85 years, present a diagnosis of MCI, Clinical Dementia Rating Scale between 0.5 and 1, HVLT-R < 5% and normal blood cell counts. Patients must also be on a stable dose of AD medication since at least 3 months.

People with diabetes, with a history of skin ulcers, in therapy with anti-platelet agents, anti-coagulant medications or other drugs affecting cytochrome CYP3A4, have been excluded. Furthermore, people with recent history of cardiovascular, major disorders, significant neurological disorders, active inflammatory, autoimmune, infectious, hepatic, malignant or psychiatric disease have been cut off. The primary completion date is estimated for July 2021. This study could be the first approach to a phase 2 clinical trial of rapamycin.

### Lenalidomide (NCT04032626)

Lenalidomide, used for multiple myeloma and myelodysplastic syndromes, acts as immunomodulator, anti-cancer and anti-angiogenic drug (Quach et al., 2010). The pleiotropic anti-inflammatory activity of the drug, combined with evidence from previous clinical trials with thalidomide, led to the construction of the study MCLENA-1 (Decourt et al., 2020) a clinical trial for the assessment of Lenalidomide in patients with MCI. The investigators designed an 18-month, Phase II, double-blind, randomized, two-armed, parallel group, placebo controlled clinical trial aimed to test the hypothesis that lenalidomide reduces inflammatory and AD-associated pathological biomarkers, thus improving cognition. Estimated enrollment counts of 30 participants, aged between 50 and 90 years with MCI diagnosed, that have been randomized into two arms: one with lenalidomide (10 mg/day orally administered for 12 months followed by 6 months of washout) and one with placebo (orally administered for 12 months followed by 6 months of washout).

Primary endpoints will evaluate the change in cognition by ADAS-Cog, ADCS-ADL, CDR-SOB, MMSE. Secondary endpoints include the AEs assessment and blood toxicity in terms of platelets falling below 50000/μL and neutrophils falling below 1000/μL. The effects on amyloid loads, CNS neurodegeneration and on blood inflammatory markers will also be assessed.

Investigators expect to first complete within September 2023. Estimated study completion date is on September 2024.

### Tacrolimus (NCT04263519)

Recent studies, which suggest a protective action of tacrolimus in countering the synaptotoxic cascade associated with Aβ (O’Neal et al., 2018), represent the basis of a phase 2, pilot, open labeled study, aimed to investigate the neurobiological effect of tacrolimus in subjects with MCI and AD-related dementia. The twelve patients enrolled have been randomized into two arms, in which, a different concentration of the drug will be collected (2–5 ng/ml vs. 5.1–10 ng/ml). Primary endpoint includes the effects of tacrolimus on CSF biomarkers (IL-2, IL-6, INFβ, YKL-40), deposition of Aβ, p-tau, and neurodegeneration. Parameters will be assessed at baseline and after 12 weeks of treatment. Effects on structural neuroimaging (MRI), electroencephalograms (EEG), on cognitive functions assessed by different inventory (MoCA, NPIQ, FAQ) will be explored as secondary outcomes. The study is planned to be completed within December 2021.

### XPro1595 (NCT03943264)

Preclinical studies have shown that selective anti-TNF biologic, XPro1595, ameliorates neurologic dysfunction in mouse models of amyloid pathology (MacPherson et al., 2017).

On the basis of such preclinical evidence, in June 2019, a multicentre phase 1b open-label trial aimed to determine the safety, tolerability, and efficacy of XPro1595 in 18 patients with mild to moderate AD and evidence of peripheral inflammation by way of elevated blood C-reactive protein has got started. Participants have received weekly injections of 0.03, 1.0, or 3.0 mg/kg XPro1595 for 12 weeks. The primary endpoint is safety, while secondary endpoints include change from baseline in biomarkers of neuroinflammation, such as blood and CSF C-reactive protein, TNF-α, interleukin-1, and interleukin-6. CSF, Aβ and tau, and cognitive and psychiatric endpoints will also be measured. The estimated study completion date is December 2020.

## Conclusion

The clinical experience gained in the arena of pharmacological treatment of inflammatory diseases represents a remarkable source of potential candidates to treat diseases with high unmet clinical need, such as AD, which may achieve considerable benefits from advantageously repositioning an array of pharmacological agents with known safety profile. Thus, unraveling inflammatory aspects of AD and compare them to mechanisms already known in other inflammatory disorders, becomes of primary relevance to reduce the disease burden in one of the most diffused dementia. In addition, the growingly shared perspective that AD not only involves activation of the immune/inflammatory response in the brain, but also depends upon peripheral immunological disturbances, helps to strengthen the concept that some of the immunomodulating drugs commonly used in inflammatory and/or proliferative diseases, might contribute to achieve meaningful clinical benefits also in AD patients. For these reasons, drug repositioning represents an appealing choice for diseases with poor therapeutic options, with the further advantage of conveniently reduced research and development costs, with special regard to clinical trials.

To date, there are no disease-modifying therapies available for AD, and the main goals of actually active trials are to detect the stage of AD at which the treatment should be more appropriately initiated, along with a durability of the treatment itself that would prevent patients from undergoing cognitive decline progression (if at all). In a clearer preclinical scenario which offers an increasing array of immune/inflammatory targets in the brain and in periphery and considering the quite wide panel of drugs which may interfere with these mechanisms, in analogy with their approved use in peripheral immune disorders, an innovative, disease modifying, treatment option(s) for AD may not be far away from the patient’s bedside.

## Author Contributions

AM and CB drafted and edited the manuscript and prepared figures and table. GD performed literature searching and drafted the manuscript. MD and RD performed searching of clinical trials. RB critically reviewed and edited the manuscript. GC conceived the idea of this review and edited the manuscript. All authors contributed to the article and approved the submitted version.

## Conflict of Interest

The authors declare that the research was conducted in the absence of any commercial or financial relationships that could be construed as a potential conflict of interest. The handling editor declared a past co-authorship with several of the authors GD, CB, RB, and GC.

## Abbreviations

AD: Alzheimer’s disease
APCs: antigen presenting cells
A β: amyloid beta
BBB: blood brain barrier
CNS: central nervous system
CSF: cerebrospinal fluid
GA: glatiramer acetate
ICR: inhibitory immune checkpoint receptor
IFN- γ: interferon gamma
IMiDs: immunomodulatory imide drugs
Mo: monocytes
MS: multiple sclerosis
mTOR: mechanistic target of rapamycin
M Φ: macrophages
NMDA: *N*-methyl -D-aspartate
PD-1: programmed cell death protein-1
PD-L1: programmed cell death ligand-1
Pom: Pomalidomide
sAPP: secreted amyloid precursor protein
TBI: traumatic brain injury
TRAIL: tumor necrosis factor-related apoptosis inducing ligand
Treg: regulatory T cells.
